# MIND Diet Intervention and Longitudinal Changes in Plasma Amyloid‐Beta 42/40 Ratio Levels in Older Adults

**DOI:** 10.1002/alz70856_104001

**Published:** 2025-12-26

**Authors:** Klodian Dhana, Konstantinos Arfanakis, Neelum T. Aggarwal, Frank Sacks, Lisa L. Barnes

**Affiliations:** ^1^ Rush University Medical Center, Chicago, IL, USA; ^2^ Department of Biomedical Engineering, Illinois Institute of Technology, Chicago, IL, USA; ^3^ Rush Alzheimer's Disease Center, Rush University Medical Center, Chicago, IL, USA; ^4^ Rush Alzheimer's Disease Center, Chicago, IL, USA; ^5^ Harvard T. H. Chan School of Public Health, Boston, MA, USA; ^6^ Department of Neurological Sciences, Rush University Medical Center, Chicago, IL, USA

## Abstract

**Background:**

Plasma amyloid‐beta (Abeta) reflects brain amyloid pathology, with a lower plasma Abeta42/40 ratio linked to a higher risk of Alzheimer's disease and related dementias (ADRD). This study investigates the effect of the Mediterranean‐DASH Intervention for Neurodegenerative Delay (MIND) diet on the longitudinal changes in plasma Abeta 42/40 levels.

**Method:**

MIND trial was a 3‐year, two‐site, randomized controlled clinical trial comparing the MIND diet with a usual diet in 604 participants aged 65 to 85 years with a family history of dementia but without cognitive impairment, being overweight and with suboptimal diets at baseline; both diets promoted weight loss through mild caloric restriction. MIND diet intervention promoted 9 brain‐healthy food groups (e.g., green leafy vegetables, nuts, berries) and limited consumption of 5 unhealthy food groups (e.g., red and processed meats, fried foods). Multivariable‐adjusted linear mixed‐effect models with a random intercept were utilized to estimate the effect of dietary interventions on the rate of change in plasma Abeta42/40 levels during the 3‐year trial period.

**Result:**

Of 604 individuals enrolled in the trial, 598 had measured plasma levels of A 42 and 40 at the baseline and 504 (84.3%) at the end of the trial (year 3). The average (SD) age at enrollment was 70.4 (4.2) years; 388 (64.9%) were female, and 524 (87.6%) were white individuals. At the baseline, Abeta42/40 levels were similar in people randomized to the MIND diet group (mean = 0.069 pg/ml) and those in the control group (mean = 0.068 pg/ml). At year 3, Abeta42/40 levels decreased in the control group while remaining statistically unchanged in the MIND diet group. Compared to the individuals in the MIND diet group, those in the control group showed an average 2.8% decline in plasma Abeta42/40 levels (beta= ‐0.012; SE = 0.005; *p*‐value=0.024) over a 3‐year follow‐up period.

**Conclusion:**

This study showed that dietary intervention influences longitudinal changes in plasma Abeta 42/40 levels. Compared to the MIND diet group, individuals in the control group showed a modest decline in plasma Abeta 42/40 levels, suggesting that the MIND diet intervention may reduce Alzheimer's disease pathology in the brain and, potentially, the risk of ADRD.

